# Aberrant right subclavian artery: a case of vertebrobasilar insufficiency

**DOI:** 10.1093/jscr/rjad199

**Published:** 2023-04-22

**Authors:** A Kimyaghalam, A Gabay, K Singh

**Affiliations:** Department of Vascular Surgery, State Island University Hospital, Staten Island, NY 10305, USA; Main Line Health, Philadelphia, PA, USA; Department of Vascular Surgery, State Island University Hospital, Staten Island, NY 10305, USA

## Abstract

Aberrant right subclavian artery (ARSA) is a rare congenital malformation, wherein the aorta gives rise to the right subclavian artery as a branch off the aortic arch distal to the takeoff of the left subclavian artery. We presented a case of a patient with ARSA that manifested vertebrobasilar symptoms. PubMed search was preformed using keywords ‘aberrant right subclavian artery’, ‘right subclavian steal’ and ‘vertebrobasilar’, which generated nine articles. We found only seven case reports through a PubMed search that discuss ARSA in association with Subclavian steal syndrome. Approximately 71% (*n* = 5) of the patients in our literature review manifested with signs and symptoms of vertebrobasilar insufficiency. Given the complex anatomy in this condition, treatment should be aimed at symptom resolution. Carotid-subclavian bypass ultimately resolved the symptoms in our patient. Management in symptomatic patient is surgical. In addition to open technique, endovascular interventions can be an option.

## INTRODUCTION

An aberrant right subclavian artery (ARSA) is a rare congenital malformation, wherein the aorta gives rise to the right subclavian artery as a branch off the aortic arch distal to the takeoff of the left subclavian artery. Although rare, an ARSA is the most common congenital anomaly of the great vessels, with a reported incidence between 0.5 and 1.8% [1]. In the majority of cases, the ARSA courses posterior to the esophagus en route to the right side of the body [2]. Nearly 60% of the time, a concurrent Kommerell’s diverticulum (KD), or an aneurysm-like dilation of the proximal opening of the ARSA, is present [3]. This diverticulum often compresses nearby structures, resulting in symptomatic dysphagia, known as ‘dysphagia lusoria’. Although dysphagia lusoria is well documented in the literature, we present a rare case of a patient with an ARSA that manifested vertebrobasilar symptoms. We also conducted a review of pertinent literature. As opposed to a KD, which is a dilation of the ARSA, our patient had severe stenosis of the ARSA at the origin, which led to retrograde blood flow through the right vertebral artery and decreased flow to the hand. The reversal of blood flow in the vertebral artery resulted in hypoperfusion to the posterior brain, which caused vertebrobasilar symptoms and claudication of the right hand and arm. Patient agreed to proceed with posting the details and images in this case report.

## METHODS

A PubMed search was preformed using keywords ‘ARSA’, ‘right subclavian steal’ and ‘vertebrobasilar’, which generated nine articles. We excluded from our search articles regarding animal experiments, articles with no association between ARSA and SSS and articles regarding aberrant left subclavian arteries.

## CASE REPORT

**Figure 1:**
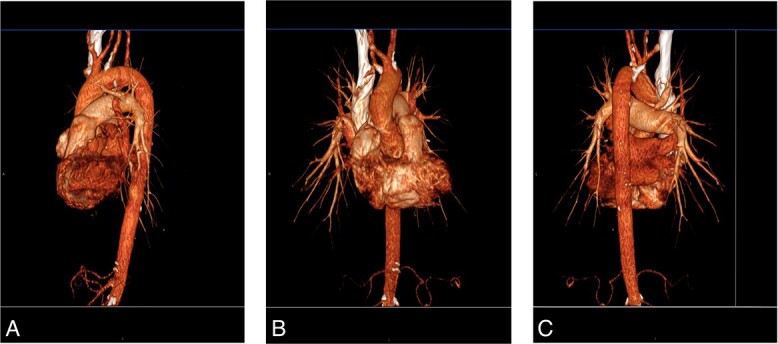
(**A**) CTA, left lateral view. (**B**) CTA, anterior view. (**C**) CTA, left posterior oblique view.

**Figure 2:**
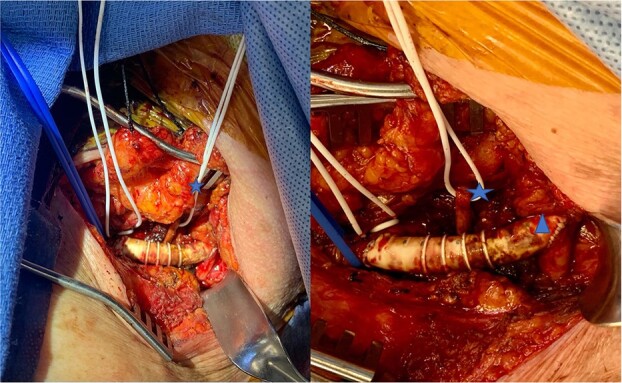
Intraoperative view of subclavian carotid bypass using PTFE graft. Blue vessel loop indicates subclavian artery to graft anastomosis. White vessel loop with blue star identifies the non-dominant vertebral artery. Blue triangle indicates graft to carotid artery anastomosis.

A 74-year-old female presented to the office with complaints of intermittent dizziness for 4 months that was progressively worsening. She also complained of right arm tingling and exertional weakness. She underwent carotid ultrasound, which showed a mild fibrocalcific plaque in the carotid bifurcation, with proximal internal carotid artery stenosis measured as 50–69%. The ultrasound also showed retrograde (oscillating) flow in the vertebral artery and diminished flow in the subclavian artery suggestive of proximal stenosis of the right subclavian artery. There was also a 40 mmHg difference compared with the contralateral brachial noninvasive pressure. Computed tomography angiography (CTA) of the chest revealed extensive calcification of the origin of the ARSA with estimated 90% diameter reduction (Fig. 1). She was offered a CSB, which she refused due to anxiety associated with undergoing an invasive procedure. After then experiencing a worsening of her aforementioned symptoms, she was again offered a bypass and again refused. To provide symptom relief, she was offered and consented to an aortogram via right radial access, which revealed a high-grade stenosis at the origin of her ARSA. Balloon angioplasty was performed with only mild angiographic improvement A stent was not placed; due to circumferential calcification, the risk of stent compression or artery rupture was considered too high. The patient only gained mild symptomatic improvement from the balloon angioplasty; definitive treatment options, namely CSB, were again discussed. The patient eventually consented to the procedure and underwent a CSB surgery utilizing a polytetrafluoroethylene (PTFE) 8 mm graft via a supraclavicular incision. End-to-side anastomosis was performed between the graft and the subclavian artery and the right common carotid artery (Fig. 2), the proximal subclavian artery was ligated proximal to the vertebral artery. Following surgery, the patient’s right arm weakness and vertebrobasilar symptoms both resolved. Additionally, she had a palpable radial pulse, which had previously been absent. The patient was discharged home on postoperative day 2. One year later, she continues to follow-up without symptoms.

## DISCUSSION

Most developmental anomalies of the great vessels are thought to come from lack of regression of certain fetal structures. Normally, the great vessels develop from a double arch system with a series of connecting vessels. During physiologic development, the right dorsal aorta will regress, leaving the left dorsal aorta to persist as the aortic arch. Physiologic atresia of the right dorsal aorta occurs distal to the right subclavian artery. Then, the right subclavian artery merges with the right common carotid artery to form the brachiocephalic trunk. However, during pathologic development of an ARSA, the right dorsal aorta will regress between the respective origins of the right subclavian artery and the right common carotid artery. Thus, these two arteries are no longer able to merge to form the brachiocephalic trunk, leaving four (instead of 3) vessels arising from the aortic arch [4].

In the late 18th century, a surgeon named David Bayford identified a 62-year-old female with an ARSA that was causing dysphagia. He named the condition ‘dysphagia lusus naturae’ or ‘freak of nature’ for the rare congenital anomaly of an ARSA that travels posteriorly, compressing either the esophagus or trachea, resulting in symptomatic dysphagia. As aforementioned, ~60% of patients with an ARSA are found to also have a KD, which is an arterial dilation near the origin of the ARSA [3]. The frequently concomitant KD can also lead to compression of the esophagus and resultant dysphagia.

In 1961, physicians Reivich, Holling, Roberts and Toole were credited as the first to report on the topic of subclavian steal syndrome (SSS) after publishing two cases of left-sided SSS [5]. Subclavian steal results from an occlusion in the subclavian artery proximal to the vertebral artery, which causes retrograde blood flow through the vertebral artery to supply the ipsilateral upper extremity. This reversal of blood flow in the vertebral artery can result in hypoperfusion to the ipsilateral posterior brain, causing vertebrobasilar symptoms such as vertigo, diplopia and presyncope. In 1986, authors Vleeschauwer, Horsch and Koln were the first to report on a case of right SSS as the result of an ARSA [6].

Although rare, ARSA associated with KD and dysphagia is well documented in the literature; however, there is a dearth of literature documenting the incidence of ARSA causing subclavian steal syndrome and vertebrobasilar symptoms. We have discussed a case presentation and we conducted a literature review and summarized our findings in Table 1.

**Table 1:** PubMed literature review.

	Patient Age/Sex	Associations	Symptoms	Imaging	KD, Stenosis or Occlusion	Treatment	Outcome
Article 1 [5]	81 M		Shortness of breath; no SSS	Carotid color Doppler US showed antegrade flow in R vertebral artery and CTA	Thrombosed KD	Anticoagulation therapy	Thrombosed KD disappeared at 1 month
Article 2 [6]	70 F	NSCLC	Incidentally found; no SSS	Reverse ipsilateral vertebral artery flow that was detected by MDCT and confirmed by color Doppler US	Thrombosed KD	Anticoagulation therapy	Unknown
Article 3 [7]	50 F		Dizziness and fainting after R arm exercise	Carotid color Doppler US showed antegrade flow in R vertebral artery and CTA	Stenosis	Stent	After surgery, the RUE pulses were restored with no difference in the systolic blood pressure between the 2 arms.
Article 4 [8]	65 F	Common trunk of the bilateral common carotid artery arose from the aortic arch and ARSA	Dizziness, numbness and weakness of the R hand	Carotid color Doppler US showed antegrade flow in R vertebral artery and CTA	Stenosis	Considering the surgical risks, the patient refused the ARSA stent and was discharged.	Unknown
Article 5 [10]	N/A		Subclavian steal symptoms		Occlusion	Right SCT	No complications at 67 months
Article 6 [11]	30 M	ARSA at site of aortic coarctation	Numbness and pain of R hand, blurred vision, and dizziness	Carotid color Doppler US showed antegrade flow in R vertebral artery and CTA	Occlusion	CSB with Dacron graft	After surgery, the patient became asymptomatic
Article 7 [12]	61 M		Syncope; SSS with loss of R arm pulses	Aortography, bilateral humeral arteriography; arteriosclerotic obstruction of the initial segment of the ARSA	Occlusion	Right SCT	Restored pulses in the R arm at 9 months postoperatively

See References [6–12]. SSS = subclavian steal syndrome; KD = Kommerell’s diverticulum; R = right; CTA = computer tomography angiography; US = ultrasound; RUE = right upper extremity; ARSA = aberrant right subclavian artery; NSCLC = non-small cell lung cancer.

We found only seven case reports through a PubMed search that discuss ARSA in association with SSS. Approximately 29% (*n* = 2) of the patients did not present with symptoms of SSS. Rather, they were incidentally found to have ARSA, and these patients were also found to have reversal of blood flow in the right vertebral artery. Of note, these same patients were the only two patients (2/7) who were found to have thrombosed KD causing the SSS. Approximately 71% (*n* = 5) of the patients in our literature review manifested with signs and symptoms of vertebrobasilar insufficiency, such as dizziness, blurry vision, syncope or weakness, in addition to the reversal of blood flow demonstrated by Doppler ultrasonography.

Initially, the patient in our case presentation was offered a carotid subclavian bypass. However, due to the invasive nature of the procedure, the patient refused. As her symptoms worsened, she continued to refuse bypass surgery, but she agreed to aortogram with angioplasty in hopes to provide symptomatic relief. Due to the luminal narrowing and severe calcification, we had difficult passing the balloon, we then switched to an 0.014 platform and performed angioplasty (5 mm balloon), the reference vessel was 7 mm. We chose a smaller balloon because of fear vessel rupture which would be difficult to control given the aberrant anatomy, ultimately and expectedly, did not provide adequate symptom relief, we avoided a stent in fear of compression and risk of over dilation in a circumferential calcified artery causing a catastrophic rupture. Eventually, she agreed to proceed with CSB surgery for definitive treatment. The bypass surgery successfully ameliorated both her vertebrobasilar and her subclavian steal symptoms. Of note, in our literature review, every patient who was diagnosed with vessel occlusion (*n* = 3) underwent surgical treatment, and each of these patients saw significant symptomatic improvement postoperatively.

Modern treatment options for an ARSA include single-stage and multi-stage procedures, such as subclavian-carotid transposition (SCT), CSB, aortic arch debranching and thoracic endovascular aortic repair [8]. These treatment options are effective for ARSA associated with KD. Treatment options for patients who have an ARSA associated with proximal stenosis without a diverticulum include SCT, CSB and balloon angioplasty with or without stent.

## CONCLUSION

ARSA is a congenital defect which most commonly results in dysphagia lusoria. Here we have presented a rare case in ARSA which led to SSS and vertebral basilar symptoms. We also reviewed literature on this rare topic and present our findings. Given the complex anatomy in this condition, treatment should be aimed at symptom resolution. A minimally invasive approach can offer temporary relief, but a CSB ultimately resolved the symptoms in our patient and offered a lasting solution to a complicated diagnosis.

## INFORMED CONSENT

Patient has given full consent for publication purposes.

## CONFLICT OF INTEREST STATEMENT

None declared.

## FUNDING

None declared.

## DATA AVAILABILITY

The authors confirm that the data supporting the findings of this study are available within the article [and/or] its supplementary materials.
